# Frequency Spectrum Method-Based Stress Analysis for Oil Pipelines in Earthquake Disaster Areas

**DOI:** 10.1371/journal.pone.0115299

**Published:** 2015-02-18

**Authors:** Xiaonan Wu, Hongfang Lu, Kun Huang, Shijuan Wu, Weibiao Qiao

**Affiliations:** 1 State Key Laboratory of Oil and Gas Reservoir Geology and Exploitation, Southwest Petroleum University, Chengdu, Sichuan, China; 2 School of Civil Engineering and Architecture, Southwest Petroleum University, Chengdu, Sichuan, China; 3 School of Petroleum Engineering, Southwest Petroleum University, Chengdu, Sichuan, China; 4 College of Pipeline and Civil Engineering, China Petroleum University, Qingdao, Shandong, China; 5 College of Petroleum Engineering, Liaoning Shihua University, Fushun, Liaoning, China; University of Aveiro, PORTUGAL

## Abstract

When a long distance oil pipeline crosses an earthquake disaster area, inertial force and strong ground motion can cause the pipeline stress to exceed the failure limit, resulting in bending and deformation failure. To date, researchers have performed limited safety analyses of oil pipelines in earthquake disaster areas that include stress analysis. Therefore, using the spectrum method and theory of one-dimensional beam units, CAESAR II is used to perform a dynamic earthquake analysis for an oil pipeline in the XX earthquake disaster area. This software is used to determine if the displacement and stress of the pipeline meet the standards when subjected to a strong earthquake. After performing the numerical analysis, the primary seismic action axial, longitudinal and horizontal displacement directions and the critical section of the pipeline can be located. Feasible project enhancement suggestions based on the analysis results are proposed. The designer is able to utilize this stress analysis method to perform an ultimate design for an oil pipeline in earthquake disaster areas; therefore, improving the safe operation of the pipeline.

## Introduction

Seismic activity is a sudden movement of the earth’s crust caused by a rapid release of earth crust energy. A seismic event is a relatively severe geological disaster, which not only destroys houses and buildings but also results in secondary disasters. In pipeline projects, an earthquake is one important cause of pipeline failure. According to statistical data provided by the Federal Emergency Management Agency (FEMA), there are two types of pipeline failure caused by earthquakes: pipeline breakage (80% of total accidents) and pipeline leakage (20% of total accidents). According to a statistical data report from the European Gas pipeline Incident data Group (EGIG), by the end of December 2005, gas pipeline fracture accidents caused by earthquakes represented 7.1% of total accidents [1–5].

There are two primary types of pipeline failure caused by an earthquake: first, the earthquake wave may cause the deformation of soil surrounding the buried pipeline, which would lead to excessive deformation of the pipeline until failure. This type of failure generally poses less of a threat to the pipeline under lower pressure, such as a water pipeline. Second, the failure is caused by the permanent deformation of the ground, which may occur during or after the earthquake, causing fault dislocations, landslides, etc.

In earthquake disaster areas, different pipeline stress analysis methods, based on different seismic resistance concepts, are used. For the relatively important pipelines, the limit-state design must be performed. The parameters for less probable earthquakes should be input, resulting in the design of a pipeline resistant to stronger earthquake action. According to GB50470 “Seismic technical code for oil and gas transmission pipeline engineering”, the seismic design of important sections of pipelines should adopt the ground motion parameters which are over 5% probability in 50 years, while pipelines that span over long distances and are less than 30m deep should be subject to ground motion parameters that are over 2% probability in 50 years [6].

For pipeline project safety, it is necessary to perform the stress analysis for oil pipelines located in seismic disaster areas. Displacement and stress caused by earthquakes can be identified based on the stress analysis, and the corresponding engineering measures can be implemented.

During the 1930’s and 40’s, researchers applied structure mechanics to analyse and solve the pipeline’s internal force [7, 8]. In order to improve the calculation accuracy, a calculation method based upon a statically indeterminate structure was used to solve the same problem and taking into account both the uniform load and concentrated load on the pipeline.

In the 1960s, the longitudinal displacement became a hot topic among researchers from different countries [9]. The former Soviet Union’s Bukhara-Ural Large Diameter Gas Transportation Pipeline’s design was based on the assumption that the gas transportation pipeline would be fully constrained by the surrounding soil. From this type of accident, experts realized that investigating the displacement and deformation pattern as well as the pipeline’s shape in the soil is necessary components of pipeline design.

In the mid-1990s, a new strength design method for pipelines was proposed, aiming to use thin-walled pipelines to reduce engineering work and decrease material costs and production expenses [10]. In researching this method, non-linear calculation was proposed, which is also accepted by ASME B31.4 and ASME B31.8, and commonly used in the United States.

In recent years, scholars have increasingly taken the stress analysis of piping which must be carried out prior to production to ensure the safety. In 2012, Wu Xiaonan proposed the tunnel pipe stress analysis model and various conditions of load combination [11]. In 2012, Huang Kun’s stress analysis model elastic laying pipelines should be used in mountainous area [12]. Since 2013, many scholars have focused special section on the pipe stress special conditions analysis, including through the swamp section, landslide area and fault area. However, there is little research about stress and displacement of oil pipelines in the seismic area [13–19].

The CAESAR II software, developed by Intergraph, has in-built stress check standards, and a variety of load working conditions can be added according to the actual situation of the project to better carry out static analysis, water hammer analysis, and fatigue analysis of the pipeline [20].

## Theory and Method

### Earthquake action

**Fig 1:**
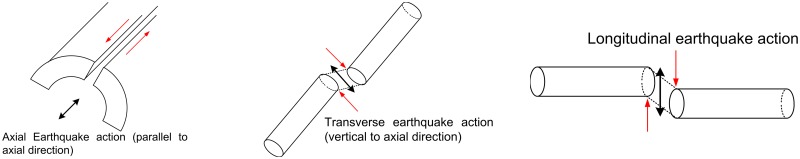
Pipeline failure mode. Pipeline failure under axial earthquake action (A), pipeline failure under transverse earthquake action (B) and pipeline failure under longitudinal earthquake action (C).

Vibrations caused by an earthquake are transmitted in the form of waves which are classified into transverse and longitudinal waves. Based on the three-dimensional model of the pipeline, transverse waves can be decomposed into those parallel and perpendicular to the pipe axis. There are three directions of earthquake action: horizontal axial, horizontal transverse and longitudinal. The failure types will vary with the different directions of earth action, which is shown in Fig. 1A, Fig. 1B, Fig. 1C.

### Loading method of seismic loads

The dynamic analysis of an earthquake is usually classified as a time history analysis or a spectrum analysis. Time history analysis is the most accurate method in the dynamic analysis of seismic engineering. Based on the methods of dynamic equations, it is a method for seeking an integral solution to structural dynamics depending on the component performance. It is suitable for solving forces that have a certain pattern, and can obtain accurately the particle acceleration, component stress, and displacement at each point of time. However, due to random variation of seismic action, it is necessary to reduce the integration step in order to obtain a more accurate calculation result, which undoubtedly increases the amount of computation [21–31]. Therefore spectral analysis is preferred in the earthquake engineering analysis.

Earthquake spectrum reflects the maximum response curve of a structure during each cycle. The spectral analysis solves the equivalent seismic action corresponding to each vibration mode guided by the principle of designing response spectrum and modal decomposition using the acceleration of a single degree of freedom system. Then certain combination principles are followed to combine the seismic effects of each vibration mode, in order to obtain seismic effects of the multiple degrees of freedom system. It not only takes into account the structural dynamic characteristics, but also converts the dynamic force problem into static force problem. Although the spectrum analysis method can only reflect the maximum elastic seismic response of the pipeline instead of changes in structural performance during the earthquake, but from the pipeline seismic point of view, the maximum seismic response obtained can meet the requirements [32–41].

Due to the uncertainty of the earthquake, the recorded results of ground acceleration are not exactly the same even in the same place and with the same seismic intensity. The most statistically representative average curve of the spectral curves that are calculated based on a large number of seismic records is called the standard spectral curve [42]. The design and value scope of the spectrum in the spectrum analysis normally refers to ASCE 7–05: *Minimum Design Loads for Buildings and Other Structures* [43].

**Fig 2:**
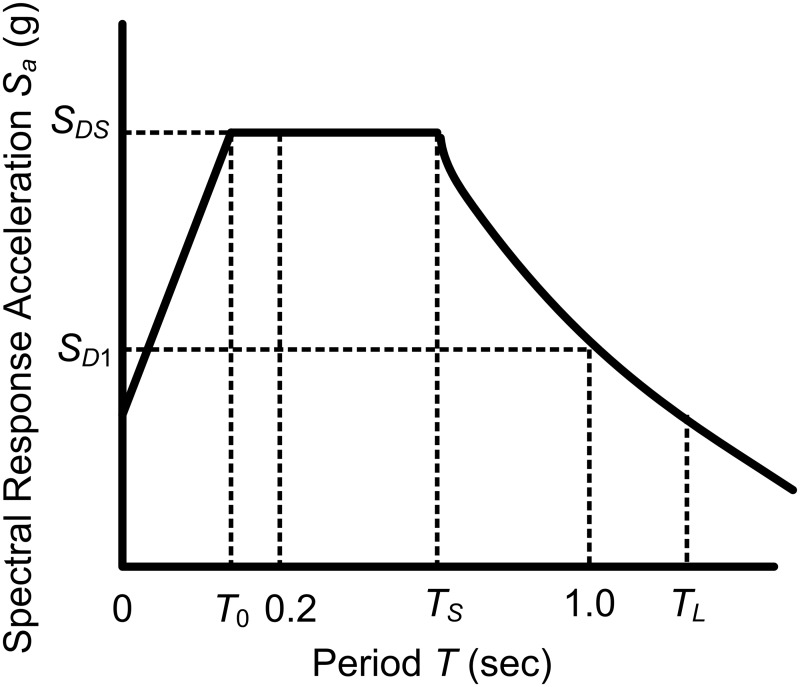
Design response spectrums.

When a design response spectrum is required by this standard and site-specific ground motion procedures are not used, the design response spectrum curve shall be developed as indicated in Fig. 2.

*S*_*DS*_ and *S*_*D*1_ are used to choose the categories and methods of pipeline seismic design. There are four cycle intervals of designed response spectrum, and different cycle intervals correspond to different spectral response acceleration calculation formulae, as shown in Table 1.

**Table 1:** Spectral response acceleration calculation formulae.

Interval	Computational formula
0≤*T*≤*T*_0_	$S_{a}=\frac{2}{3}F_{a}S_{s}(0.4+0.6\frac{T_{p}}{T_{0}})$
*T*_0_≤*T*≤*T*_*s*_	$S_{a}=\frac{2}{3}F_{a}S_{s}$
*T*_*s*_≤*T*≤*T*_*L*_	$S_{a}=\frac{2F_{v}S_{1}}{3T_{p}}$
*T*_*L*_≤*T*	$S_{a}=\frac{2F_{v}S_{1}T_{L}}{3T_{p}^{2}}$

*S*_*a*_: Spectral acceleration, g (g is the local gravitational acceleration, with the unit of m/s^2^); *S*_*S*_: Spectral acceleration for short cycles (damping factor is 5%); *S*_*1*_: Spectral acceleration with the cycle as 1s (damping factor is 5%); *F*_*a*_, *F*_*v*_: Venue coefficient; *T*_*p*_: Inherent cycle of a structure; *T*_*0*_: Cycle of response spectral characteristics, *T*_*0*_ = 0.2*S*_*D*1_/*S*_*DS*_; *T*_*s*_: Cycle of response spectrum characteristics *T*_*s*_ = *S*_*D1*_/*S*_*DS*_; *T*_*L*_: Transitional period of long cycle; *S*_*DS*_: Design spectral response acceleration for short cycle (damping factor is 5%); *S*_*D1*_: Design spectral acceleration with the cycle of 1s (damping factor is 5%).

### Stress analysis method

The finite element is a commonly used method in pipe stress analysis. Different from softwares such as ANSYS, CAESAR II reflects pipe stress and displacement by calculating the pipeline unit endpoint, and the method is simpler to add constraints or load.

The analysis of long-distance pipeline stress in the earthquake region is generally divided into two modules: (1) static analysis and (2) dynamic analysis. Static analysis is the basis of stress analysis. Its main purpose is to analyse the distribution of stress or displacement of the pipe in the absence of seismic conditions. Dynamic analysis is used to analyse the stress and displacement of the pipeline under the earthquake situation. For pipelines in special terrains (such as those crossing earthquake zones, landslide areas and swamps), usually both modules are needed in the analysis.

**1. Static stress analysis**. The main content of static analysis is to establish the corresponding model in accordance with the pipeline construction plans, and to determine the combination type of the working conditions based on pipeline loads, whereby the static analysis is performed.

Establishment of the pipeline system: the basic pipeline parameters (i.e., pipeline diameter, pipeline thickness, materials, etc.) and the environmental parameters (i.e., pipeline operating temperature and pressure) must be input into the operational interface.

Establishment of constraints: based on engineering practice, constraints must be simplified and then input into the operational interface.

Establishment of load cases: because the medium inside the pipeline and its environment are different at different stages, the load cases must be established based on the different loads from production to operation.

Exporting analysis results: the static stress analysis report is generated from the software calculations.

**2. Dynamic earthquake analysis**. Depending on the load characteristics of the earthquake zone, dynamic analysis is adding seismic effects on the pipe to make it as close as possible to the actual project. By calculating the various ground motion parameters, the calculated acceleration will be transformed into seismic effects and added onto the basic pipe model, enabling dynamic analysis of the pipeline to be performed.

Define the frequency spectrum: input the earthquake related coefficients and CAESAR II can generate the corresponding frequency spectrogram.

Spectrum loading: define the loading coefficients, direction, and the seismically affected start and end points of the pipeline.

Dynamic load condition combination: the loads are combined and loaded in the pipeline model.

### Pipeline mechanics model

Pipe stress analysis is based on the finite element method, and the finite element models can generally be divided into the beam model and the shell model. As the pipeline itself is an axisymmetric closed cylindrical shell, the shell model for analysis would be more accurate. However, in order to save computer resources, usually 3D beam element model is adopted. This is because in an earthquake area, the length of the pipe is much larger than its diameter, and the major consideration is the bending deformation rather than the deformation and stress within the pipe cross-section. Long-term engineering practice has also shown that for pipes with the diameter no more than 100, using beam element model can meet the computational accuracy [17].

**Fig 3:**
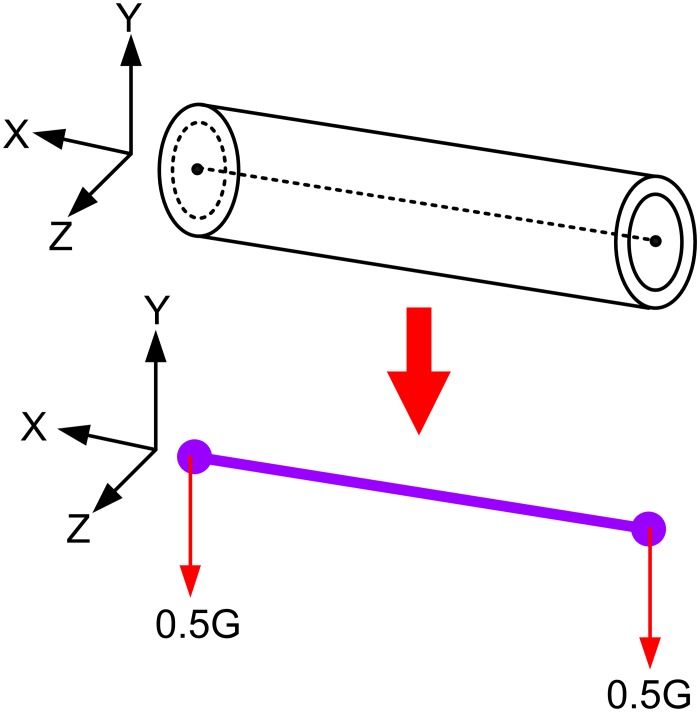
3D beam element model.

As shown in Fig. 3, the 3D beam element model’s simulation of the pipeline is a rigid element with six degrees of freedom. The mechanical behaviour of each unit (each pipe section) is described with the endpoints, i.e., the objects of stress calculation are the two ends of a pipe unit, and the weight of the pipe is evenly distributed to both ends of the pipe unit [20, 44]. The mechanical hypothesis of the 3D beam unit is as follows:

1. The pipe is a pure bending deformation, which obeys Hooke’s law;
2. Ignore the effects of shear stress;
3. Constraint effects are on the centre line of the unit.

### Soil mechanics model

Long-distance transmission pipelines are mostly laid under the ground, and for underground pipelines, the most important constraint comes from the soil. Soil constraints on the pipeline are mainly two types. One is friction: the sudden slide of the pipeline needs to overcome the friction; the other is the pressure, which is produced by the pipeline’s push of the soil. Soil not only constrains the axial, lateral and longitudinal movement of the pipe, but also bears the weight of the pipe. Under real conditions, soil deformation and changes in constraints are nearly linear, and the greatest soil constraints appear when pulled and compressed [45–49].

**Fig 4:**
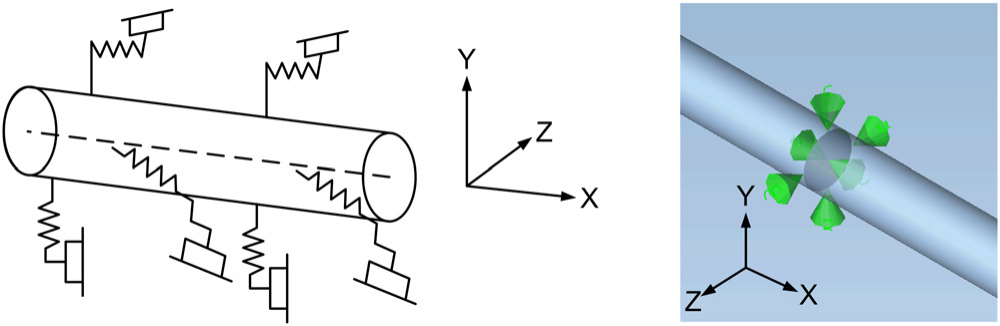
Soil model. (A) Soil Constraints and (B) soil numerical simulation model.

In order to facilitate the analysis of piping stress using the finite element method, usually continuous soil will be discrete into three unidirectional springs with bilinear stiffness, as shown in Fig. 4A, soil numerical simulation model is shown in Fig. 4B. Soil spring stiffness approximates its true deformation—constraint curve slope, but critical condition is the maximum soil constraint. Peng is a currently commonly used model for solving the soil stiffness and the maximum constraint [50].

In pipe stress analysis, three more common soil forces are horizontal, longitudinal and axial forces. The effect of each lateral resistance can be reduced to continuous action stages. One of them is the elastic stage, where the resistance is proportional to the pipe displacement, and the other is the viscous stage, where no matter how much the displacement is, the resistance is constant. This type of constraint can be modelled as a bilinear constraint requiring elastic stiffness, ultimate load (transition from elastic to viscous) and stick stiffness (should be close to 0). Soil not only limits the movement of the pipe, but also limits its rotation through the couple [50].

### Boundary conditions

In order to prevent bending caused by the weight of the entire pipeline system, fixed piers are installed to eliminate the effects of the pipeline outside the model on the pipeline. The displacements of the pipeline on both sides of the fixed piers are independent of each other, and the change in stress cannot be transmitted through them. In practical applications, fixed piers are mitered, and deviate from adjacent joints of the pipeline system. Therefore, fixed piers are constrained from displacing and bearing axial forces, but they can bear bending moments and shear forces [17].

### Stress calibration methods

The CAESAR II software allows for the selection of different calibration standards for different research objects. Because this research involves an oil pipeline crossing an earthquake disaster area, the software adheres to the calibration method from ASME B31.4 *Pipeline Transportation Systems for Liquids and Slurries* [51]. The stress calibration of an oil pipeline in an earthquake disaster area includes dynamic and static loads, and their calibration bases are different. The displacement calibration is based on the requirement of GB50253 *Code for design for oil transportation pipeline engineering* [52].

**1. Static calibration**. The static stress calibration of an oil transportation pipeline in an earthquake disaster area considers a sustained load (axial stress) condition; the axial stress reflects the effects of self-weight, a medium weight inside the pipe and the internal pressure on the pipeline. In the CAESAR II software, this sustained load is usually expressed as [SUS] = *W*+*P* (W is self-weight, *P* is pressure load).

Based on the term 402.3.1 in ASME standard B31.4：For the restricted pipeline, *S*_*H*_ should be smaller than 0.72 times the yield strength, which is *S*_*H*_ < 0.72*SMYS*, therefore:

$$S_{H}=\frac{PD}{20t}$$ (1)

where *D* is pipe outside diameter, mm; *P* is the design pressure for the pipeline, bar; *S*_*H*_ is the hoop stress from the internal pressure of the pipeline, MPa; *t* is the pipe thickness, mm; and *SMYS* is the minimum yield stress of pipeline, MPa.

**2. Dynamic calibration**. The dynamic stress calibration of an oil transportation pipeline focuses on the incidental load condition. In the Cartesian coordinate system, the direction of earthquake load is divided into the following three directions: axial (X direction), longitudinal (Y direction) and transverse (Z direction). Their calibration bases are same. In CAESAR II, the dynamic load condition of an earthquake is normally expressed as [OCC] = *W+P+D* (*D* is the dynamic load).

Based on the term 402.3.1 in ASME standard B31.4: The sum of the longitudinal stresses produced by pressure, live and dead loads, and those produced by occasional loads, such as wind or earthquake, shall not exceed 80% of the specified minimum yield strength of the pipe, which is *S*_*L*_ ≤ 0.80*SMYS*, therefore:

$$S_{L}=E\alpha (T_{2}-T_{1})-\nu S_{h}$$ (2)

where *S*_*L*_ is longitudinal stresses, MPa; *SMYS* is the minimum yield strength of the pipe, MPa; *E* is the modulus of elasticity of steel, MPa; *S*_*h*_ is the hoop stress from fluid pressure, MPa; *T*_*1*_ is the temperature at time of installation, °C; *T*_*2*_ is the maximum or minimum operating temperature, °C; *α* is the linear coefficient of thermal expansion, mm/mm°C; *ν* is Poisson’s ratio (*ν* = 0.30 for steel).

**3. Displacement calibration**. There is no accurate standard for the pipeline displacement calibration. In section 5.6.2 of GB50253 *Code for design for oil transportation pipeline engineering*, there is a general specification for the displacement condition of a steel pipeline: the maximum displacement of a steel pipeline in the horizontal direction should not exceed 0.03 times the average diameter of the steel pipeline, which is

Δx≤0.03D (3)

where Δ*x* is the maximum displacement of the steel pipeline in the horizontal direction, m; and *D* is the average diameter of the steel pipeline, m.

## Case Study

### Project introduction

**Fig 5:**
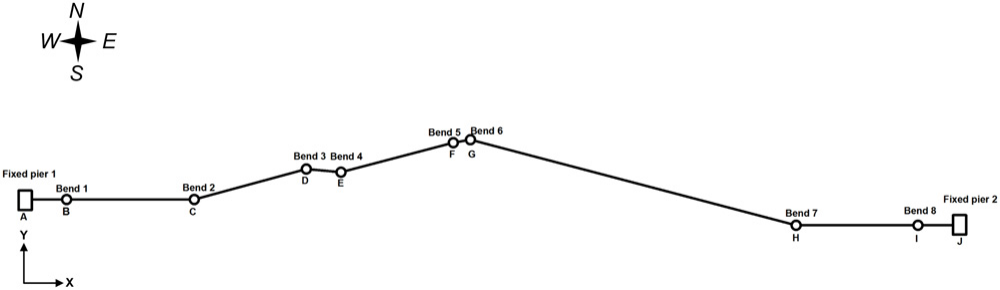
Spatial layout of a pipeline in an earthquake disaster area.

Based on the design materials for the XX earthquake disaster location, X80 steel pipe is used for the oil pipeline, the installation temperature is 20°C, the running temperature is 80°C and the operating pressure is 8 MPa. The ambient temperature of the pipeline is 20°C, the backfill soil is saturated coarse sand and the seismic intensity level is 7, (a low probability earthquake for limit-state design consideration). The basic seismic acceleration is 0.30 g, the elevation of the bottom slope at the west side of the pipeline is 361.66 m, the elevation of the middle slope is 383.95 m and the elevation of the top slope is 408.33 m. The direction of the pipeline is shown as Fig. 5. The detailed parameters of the pipeline layout are shown in Table 2. The specific pipeline, soil and earthquake parameters are shown in Tables 3–5.

**Table 2:** Special location parameters of pipelines.

Azimuth	Pipe Segment	Axial Deviation (°)	Longitudinal Grade (°)	Remarks
West Side	AB	30	0	A is the pipe’s starting point, that is anchor block 1
	BC	0	0	B is the guiding point for the west side pipe
	CD	0–15	15	——
	DE	0–10	5	——
	EF	0–15	15	——
	FG	0–10	10	——
East Side	GH	0–15	15	——
	HI	0	0	I is the guiding point for the east side pipe
	IJ	30	0	J is the pipe’s end point, which is anchor block 2

**Table 3:** Pipeline parameters.

Material	Diameter (mm)	Thickness (mm)	Corrosion allowance (mm)	Thickness of insulation layer (mm)
X80	406	7.1	1	60

Density of oil (kg·m^-3^)	Installation temperature (°C)	Operation temp (°C)	Operation pressure (MPa)	Minimum yield strength (MPa)
900	20	80	8	551.58

**Table 4:** Soil parameters.

Type	Friction coefficient	Density (kg·m^-3^)	Temperature variation (°C)
Dried coarse sand	0.6	2600	60

Soil Friction Angle (°)	Compaction coefficient	Thermal expansion coefficient	Yield Displacement Factor (L/L/°C)
30	5	11.214×10^–6^	0.015

**Table 5:** Seismic Parameters.

Seismic impact	Seismic Type	Basic seismic accretion	Maximum value of horizontal seismic affecting factor
Level 7	Rare	0.30 g	0.72

The pipe is a spatial model, i.e., there are the length components in all three directions of X, Y, Z. The total length of the pipeline model is 780 m. fixed piers 1 and 2 are set at the start and end points, respectively, of the pipeline. There is 30 m of pipeline before the west side soil entrance (guidance point B), and the entrance angle is 30°. The west horizontal section is 110 m and the inclined pipeline is 215 m. There is 30 m of pipeline after the east exit (guidance point I), and the exit angle is 30°. The east horizontal section is 105 m and the east inclined pipeline is 290 m. The curvature radius of the bended pipeline is *R* = 10*D*, in which *D* is the outside diameter [16].

### Earthquake analysis and loading

Based on the earthquake related materials for the XX pipeline and the GB50011–2010 Code for seismic design of buildings, the ground’s characteristic period and the maximum value of the horizontal seismic coefficient can be obtained. According to Formulas (4) and (5), the response acceleration *S*_*S*_ of the short period design spectrum of the damping coefficient is 4% (the U.S. nuclear pipeline design manual requires that the damping coefficient equals 4%) and the response acceleration *S*_1_ of the design spectrum with a one second period is calculated. Based on Tables 11.4–1 and 11.4–2, from ASCE 7–05, the site coefficients *F*_*a*_ and *F*_*v*_ are determined. After inputting the related earthquake frequency spectrum parameters into the frequency spectrum analysis modules, the analysis chart will be generated by the software. The frequency spectrum parameters of the XX pipeline are shown in Table 6.

$$S_{S}=1.604\alpha_{\max}/F_{a}$$ (4)

$$S_{1}=1.604T_{g}^{0.918}\alpha_{\max}/F_{v}$$ (5)

**Table 6:** Spectrum Parameters.

Component significant coefficient	Land coefficient		*S_S_*	*S_1_*	Modified response coefficient
	*F_a_*	*F_v_*			
1.5	1.0	1.3	1.24	0.70	3.5

where *α*_max_ is the maximum value of the horizontal earthquake affecting factor, 0.72 in this case; and *T*_*g*_ is the characteristic period of the ground.

After loading the spectrum to the pipeline model, the earthquake working conditions must be established. We analysed one static working condition and four dynamic working conditions. The dynamic condition comprises occasional load condition in each of the axial direction (X direction), the longitudinal direction (Y direction) and the transverse direction (Z direction), as well as the occasional load condition with three directions combined with the stress checking method identical to one-direction occasional load conditions. Of particular note is that the combined occasional load condition is established through transforming into seismic action the acceleration calculated based on the spectrum in three directions, and loading the seismic action on the pipeline by combining load conditions. The integrated seismic acceleration (force) is calculated following acceleration (force) synthesis theorem in the Cartesian coordinate system. The working condition combinations are listed Table 7.

**Table 7:** Instruction for working condition combinations.

Load Type	Working condition	Express in CAESAR II	Remarks
Static	Sustained static condition	*W+P*	Regular primary stress
Dynamic	Loading the seismic action condition in X direction	*W+P+D*_X_	Seismic occasional stress in X direction
	Loading the seismic action condition in Y direction	*W+P+D*_Y_	Seismic occasional stress in Y direction
	Loading the seismic action condition in Z direction	*W+P+D*_Z_	Seismic occasional stress in Z direction
	seismic action condition of three combined directions	*W+P+D*_X_ *+D*_Y_ *+D*_Z_	Seismic occasional stress of three combined directions

*D*_X_: Seismic action in X direction; *D*_Y_: Seismic action in Y direction; *D*_Z_: Seismic action in Z direction.

## Results

### Numerical Simulation result

After building the dynamic load and combining the working conditions, the CAESAR II software can produce the dynamic stress analysis report, which includes the details of the pipeline stress, displacement, constraints loading, etc. Because the pipeline start points, end points, west side guidance section and east side guidance section aim to reduce the influence from the pipeline located outside the model, when performing the stress analysis, the displacement and stress of these four special locations can be neglected in the XX earthquake disaster section. Consequently, the pipeline is studied from 32 m-740 m (I.e., from point B to point I, and not including bends 1 and 8).

After gathering the maximum displacement and stress (Tables 8 and 9), in the comprehensive seismic load condition, the maximum axial, longitudinal displacement and transverse displacement are generated at the bend in the pipeline on the top of the slope (bend 6). The following conclusions can be drawn after calibrating the static stress, dynamic stress and displacement: the maximum stress ratio does not exceed calibrated stress, which meets the strength requirement; and the maximum transverse displacement does not exceed 0.03*D* = 12.2 mm, which meets the displacement requirement. Comparing the maximum axial displacement and the longitudinal displacement during seismic activity, the maximum transverse displacement is larger. Therefore, in actual projects, it is suggested to add the corresponding pipeline parts necessary to restrain the transverse displacement of the pipeline.

**Table 8:** Pipeline stress calibration under static and dynamic conditions.

Type	Direction of seismic action	Maximum Stress (MPa)	Location	Calibrated Stress (MPa)
Static Stress	——	100.00	Bend 3	397.14
Dynamic Stress	X	204.17	Bend 4	441.26
	Y	201.74	Bend 4	441.26
	Z	204.52	Bend 4	441.26
	Combination of three directions	229.94	Bend 4	441.26

**Table 9:** Displacement calibration of pipeline under static and dynamic working conditions.

Type	Seismic action direction	Maximum axial displacement (absolute)		Maximum longitudinal displacement (absolute)		Maximum transverse displacement (absolute)		Calibrated Displacement (mm)
		Displacement/mm	Location	Displacement/mm	Location	Displacement/mm	Location	
Static	——	1.50	Bend 6	1.98	Bend 6	2.95	Bend 6	12.2
Dynamic	X	0.41	Bend 6	0.11	Bend 6	0.11	Bend 6	12.2
	Y	0.05	Bend 2	0.03	Bend 7	0.03	Bend 7	12.2
	Z	0.04	Bend 2	0.03	Bend 6	0.03	Bend 6	12.2
	Combination of three directions	1.92	Bend 6	2.05	Bend 6	1.69	Bend 6	12.2

**Fig 6:**
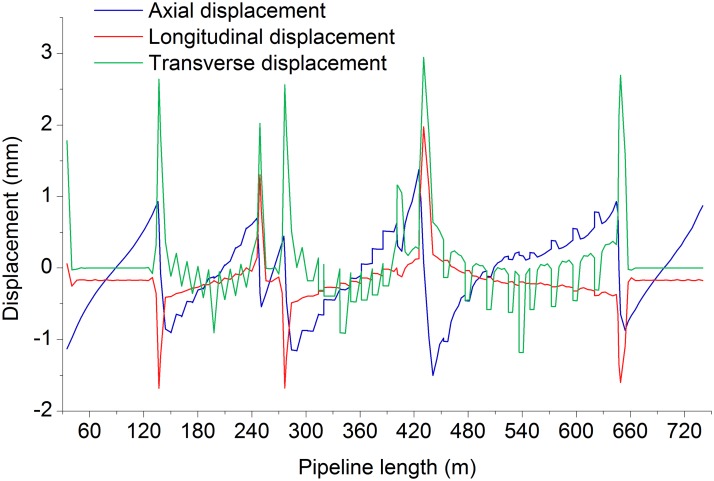
Distribution of displacement in axial, longitudinal and transverse directions of static pipeline.

**Fig 7:**
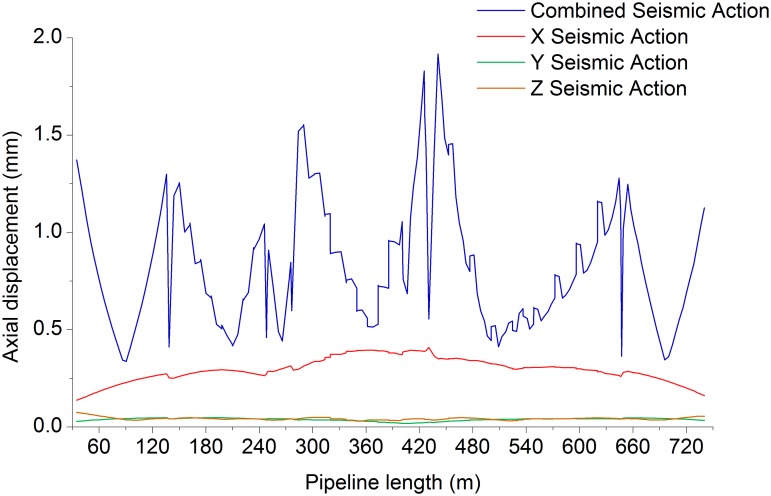
Pipeline axial displacement during seismic action.

**Fig 8:**
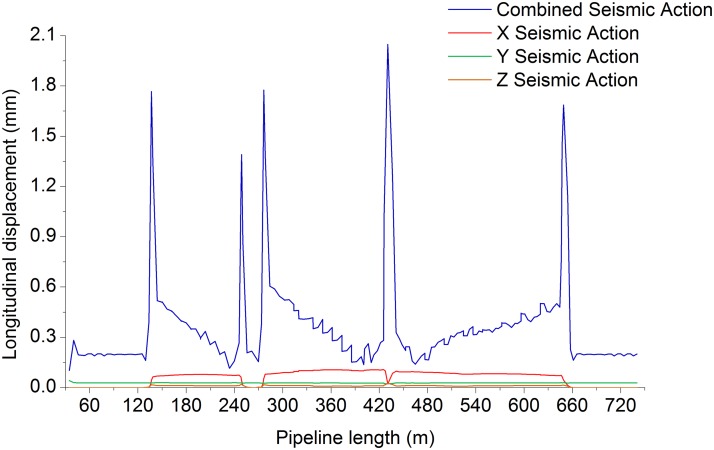
Pipeline longitudinal displacement during seismic action.

**Fig 9:**
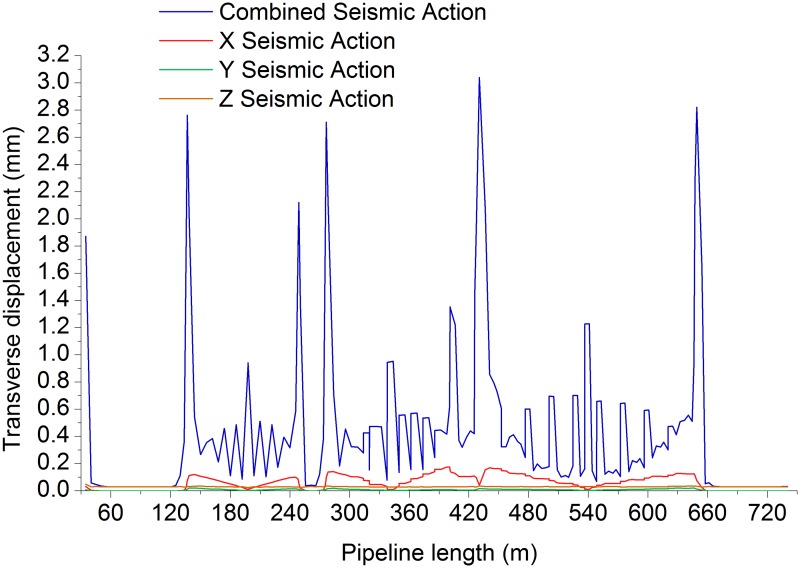
Pipeline transverse displacement during seismic action.

**Fig 10:**
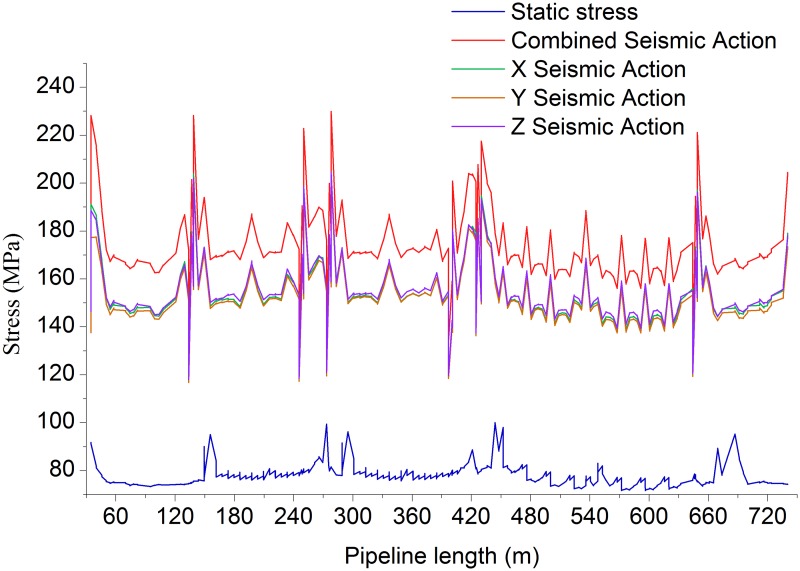
Pipeline stress distribution (Static and dynamic).

Figs. 6–9 shows the axial, longitudinal and transverse displacement distributions along the pipeline under static conditions and earthquake activity, respectively. The displacement during earthquake activity is calculated using the SRSS (Square Root of the Sum of the Squares) method [53–60] (This is one of the many types of modal combination method. This method assumes that events involved in data processing are completely independent of each other. When the difference between natural vibration states of a structure or between the natural vibration frequencies is large, it can be approximately considered that the vibration of each mode is independent of each other, so that a better result can be obtained, but the symbol of the vibration model cannot be displayed.). All calculated values are positive, which indicates the pipeline’s maximum distance at a certain moment but does not show the movement direction; therefore, the static displacement situation has been presented separately. Fig. 10 displays the distribution of stress ratios along the pipeline under static conditions and earthquake activity. Based on Figs. 6–10, the following can be concluded:

1. Compared to the transverse and longitudinal earthquake action, the axial (X direction) earthquake action causes greater changes to the axial, longitudinal and transverse displacements, which suggests the necessity to include axial earthquake action controls into the design.
2. The pipeline stress distribution trends during earthquake activity and static conditions exhibit a high degree of unity; the stress value under dynamic earthquake activity is relatively greater. For earthquake action in a single direction, the transverse earthquake activity effect is slightly larger than the effects of the earthquake activity in the other two directions
3. Under combined seismic activity, the displacement and stress of a pipeline are greater than any one single direction seismic activity, indicating that the combined earthquake activity causes the greatest damage to the pipeline.
4. The maximum displacement value and maximum stress are generated at the bent pipe on the top of the slope and on the bottom of the west slope; the stress value at the bent pipe changes suddenly. Consequently, during oil pipeline operation, there is a stress concentration at the bend location, which is the critical section of the oil pipeline in an earthquake disaster area. The stress calibrations for the bent pipeline at the top and bottom of the slope must be calculated. If the stress and displacement exceed the limits, a reinforcement measure must be implemented.
5. Stress analysis usually occurs before the pipeline is put into production, and the objects of the analysis are new steel pipes. In actual engineering, there may be cracks on the pipe due to construction and other reasons. Based on the analysis results, we can conclude that the earthquake will exacerbate cracks in the pipe in varying degrees. As the earthquake has significant impact on the lateral displacement of the pipeline, it means that the earthquake has great impact on the extension of cracks along the axial direction of the pipeline. It is therefore proposed that in oil pipeline defect detection in earthquake regions, attention should be paid to issues such as crack repair.

### Experiment result

In order to verify the correctness of the numerical simulation results and the reasonableness of the model, field experiments were conducted and the results were compared with numerical simulation results. As the test system, which includes the work strain gauges, compensation strain gages, strain gauges and computers installed on the multiple sections of the pipe, had been installed before the pipeline was put into production, we selected earlier recorded results of stress and strain with the seismic intensity of 7. Because the pipeline was long, we only selected data at the section of critical stress (bends 2 to 7), and compared them with the numerical simulation results. Relative error analysis (two decimal places) of the numerical simulation and field experimental data is calculated as follows:

$$\text{Relative error=}\frac{\text{Numerical simulation data-Experimental data}}{\text{Experimental data}}\times 100\text{\%}$$ (6)

The main factors leading to the experimental measurement error were: wire resistance, temperature, sensitivity coefficient, and moisture of strain gauges. Since the annual rainfall in the area is relatively small, the environment relatively dry, and the impact of wire resistance, temperature and the sensitivity coefficients on the measurement relatively small, they were negligible. Error analysis results showed that the range of the absolute value of the relative error of the stress test was 0.95%-3.09%, and the absolute value range of the relative error of the displacement was 0.51%-4.49%, within the acceptable range. This suggested that numerical simulation data had a certain degree of credibility, and the model was relatively reasonable.

## Discussions

### The impact of the bend angles

Oil transmission pipelines adopt the elastic laying method, that is, when the line direction changes, the spatial direction of the pipe can be changed relying on the gravity and elastic bending, without on-site installation of cold simmer bends. Different bend angles have different impacts on stress and displacement. In this real engineering case, the bend angle range was 25°- 45°. Based on the stress analysis report, we obtained the stresses and displacements of the bends (bends 2 to 7) under different working conditions.

**Fig 11:**
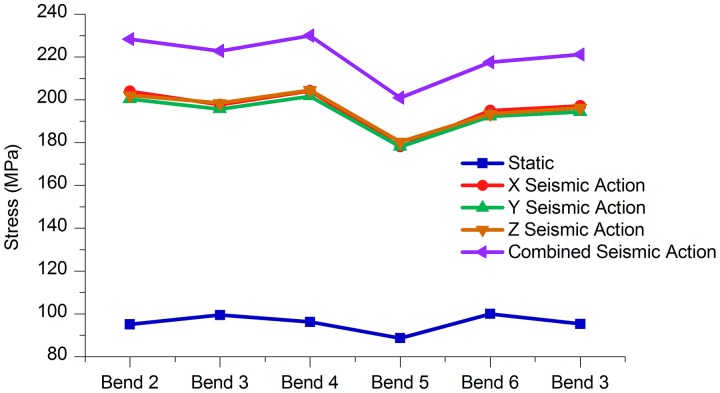
Stress of each bend under different working conditions.

As can be seen from Fig. 11 and Table 10, stress at bend 5 (25°) under various working conditions was the least, but those at bend 4 (35°) and bend 6 (45°) were larger, and the trends of stress changes of the different bends under different working conditions were highly similar. The stress value of bend 4 was 30MPa higher than that of bend 5 under the integrated seismic effects, while the stress value of bend 6 was approximately 12MPa higher than that at bend 5 in static load cases.

**Table 10:** Comparison of stresses of bends (25°–45°) under different working conditions.

Bend (Angle)	Static condition	X Seismic Action condition	Y Seismic Action condition	Z Seismic Action condition	Combined Seismic Action condition
Bend 2 (30°)	95.04 MPa	203.95 MPa	200.46 MPa	202.05 MPa	228.22 MPa
Bend 3 (35°)	99.4 MPa	197.77 MPa	195.65 MPa	198.52 MPa	222.83 MPa
Bend 4 (35°)	96.19 MPa	204.17 MPa	201.74 MPa	204.52 MPa	229.94 MPa
Bend 5 (25°)	88.72 MPa	178.18 MPa	178.18 MPa	180.43 MPa	200.90 MPa
Bend 6 (45°)	100.00 MPa	194.86 MPa	192.26 MPa	193.45 MPa	217.56 MPa
Bend 7 (30°)	95.27 MPa	197.15 MPa	194.33 MPa	196.27 MPa	221.11 MPa

**Fig 12:**
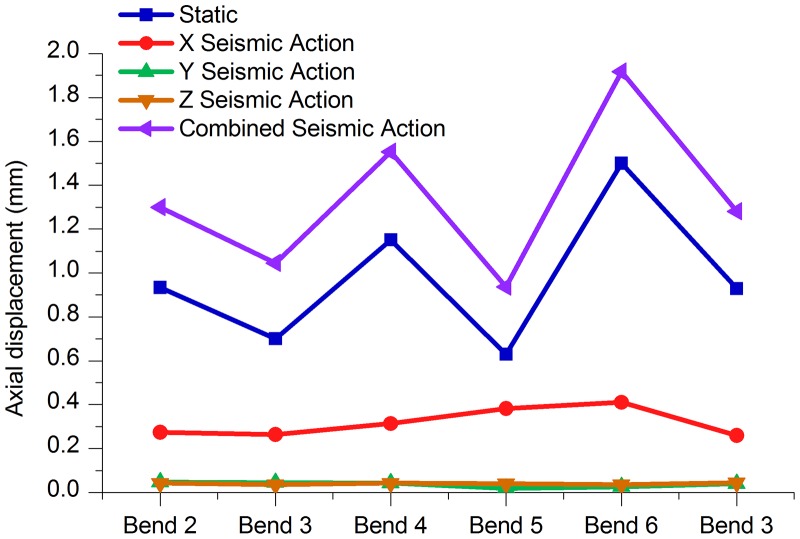
Axial Displacement of each bend under different working conditions.

**Fig 13:**
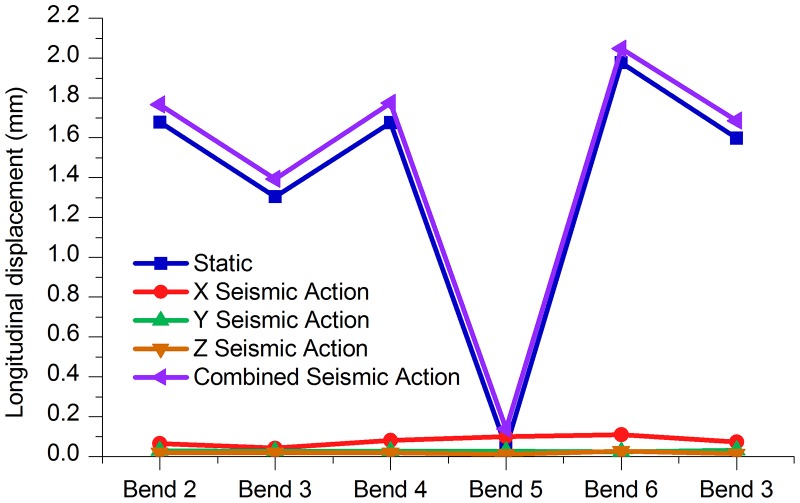
Longitudinal displacement of each bend under different working conditions.

**Fig 14:**
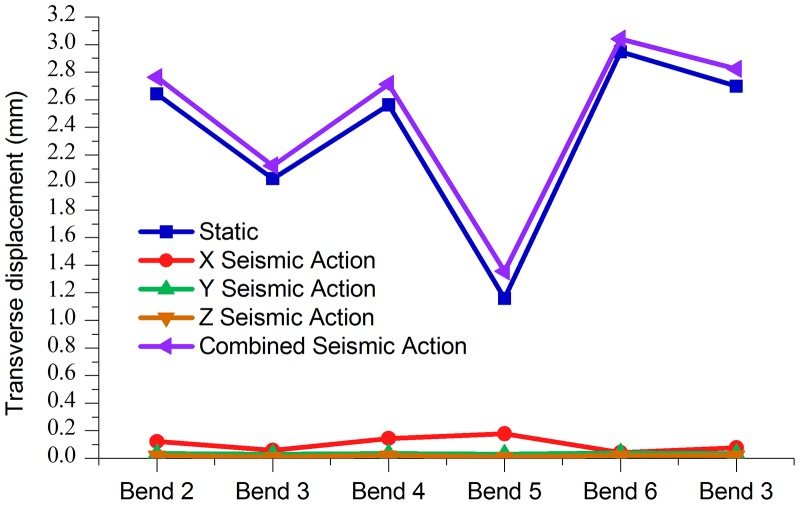
Lateral displacement of each bend under different working conditions.

Tables 11 to 13 and Figs. 12–14 indicate that, under the static conditions and integrated seismic effect working conditions, the axial displacement, the longitudinal displacement and lateral displacement of bend 6 were all higher than those of the other bends, while the displacement values of bend 5 were below the other bends.

**Table 11:** Comparison of axial displacements of bends (25°–45°) under different conditions.

Bend (Angle)	Static condition	X Seismic Action condition	Y Seismic Action condition	Z Seismic Action condition	Combined Seismic Action condition
Bend 2 (30°)	0.933 mm	0.273 mm	0.048 mm	0.043 mm	1.299 mm
Bend 3 (35°)	0.701 mm	0.264 mm	0.044 mm	0.036 mm	1.044 mm
Bend 4 (35°)	1.152 mm	0.314 mm	0.043 mm	0.043 mm	1.553 mm
Bend 5 (25°)	0.629 mm	0.383 mm	0.020 mm	0.039 mm	0.936 mm
Bend 6 (45°)	1.500 mm	0.410 mm	0.025 mm	0.035 mm	1.917 mm
Bend 7 (30°)	0.930 mm	0.260 mm	0.041 mm	0.044 mm	1.280 mm

**Table 12:** Comparison of longitudinal displacements of different bends (25°–45°) under different working conditions.

Bend (Angle)	Static condition	X Seismic Action condition	Y Seismic Action condition	Z Seismic Action condition	Combined Seismic Action condition
Bend 2 (30°)	1.678 mm	0.065 mm	0.029 mm	0.017 mm	1.767 mm
Bend 3 (35°)	1.304 mm	0.041 mm	0.027 mm	0.018 mm	1.391 mm
Bend 4 (35°)	1.676 mm	0.080 mm	0.027 mm	0.017 mm	1.775 mm
Bend 5 (25°)	0.048 mm	0.100 mm	0.027 mm	0.010 mm	0.138 mm
Bend 6 (45°)	1.977 mm	0.108 mm	0.023 mm	0.026 mm	2.048 mm
Bend 7 (30°)	1.598 mm	0.073 mm	0.030 mm	0.013 mm	1.686 mm

**Table 13:** Comparison of lateral displacements of bends (25°–45°) under different conditions.

Bend (Angle)	Static condition	X Seismic Action condition	Y Seismic Action condition	Z Seismic Action condition	Combined Seismic Action condition
Bend 2 (30°)	2.640 mm	0.120 mm	0.034 mm	0.020 mm	2.762 mm
Bend 3 (35°)	2.026 mm	0.055 mm	0.031 mm	0.006 mm	2.120 mm
Bend 4 (35°)	2.564 mm	0.142 mm	0.035 mm	0.018 mm	2.712 mm
Bend 5 (25°)	1.157 mm	0.176 mm	0.030 mm	0.009 mm	1.353 mm
Bend 6 (45°)	2.947 mm	0.041 mm	0.036 mm	0.017 mm	3.040 mm
Bend 7 (30°)	2.697 mm	0.076 mm	0.035 mm	0.019 mm	2.821 mm

**Fig 15:**
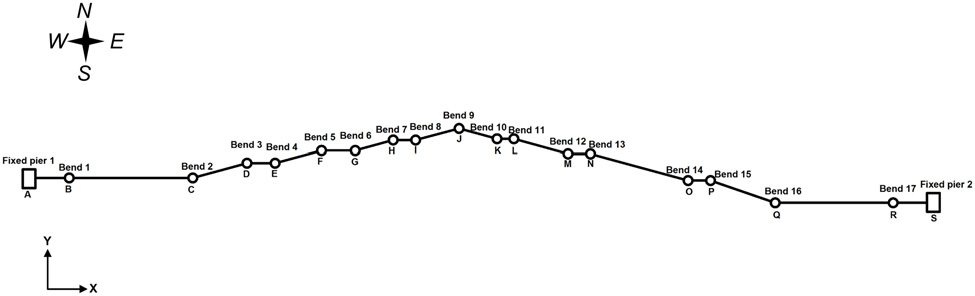
"Ladder" pipe laying schematic model.

Through the above analysis, we have come to the conclusion that the bend angle had varying degrees of impact on the stress value and displacement value. In order to ensure the safe operation of the oil pipeline during the earthquake, we propose that at the stage of pipeline design, bend angle should be reduced as much as possible. In addition, in order to effectively reduce the bend angle, the “ladder” laying (laying diagram as shown in the diagram in Fig. 15) can be introduced. This not only can reduce slope length, increase the slope waist length, and effectively reduces the bend angle, but also can effectively reduce the total elevation difference of the pipe.

### The impact of soil compaction

An earthquake makes damage to a pipeline mainly due to its impact on the soil, which in turn compresses the pipe. Therefore, for an oil pipeline which has been built, we hypothesized that reducing the degree of compaction of the soil could reduce the impact of earthquakes on the stress and displacement of the pipe.

In the CAESAR II software, the different degree of soil compaction was achieved by changing the value of Compaction coefficient. To verify whether the hypothesis was correct or not, we changed the value of the compaction coefficient in the soil parameters (the original value was 5, as shown in Table 4) on the basis of the original pipe model and integrated seismic loading conditions, and analysed the stresses and displacements of bends, respectively, when the soil compaction coefficients in the soil were 1–4. To simplify the analysis process, we only took the average pipe stress as well as the stress and displacement at the bends as the object of the study.

**Fig 16:**
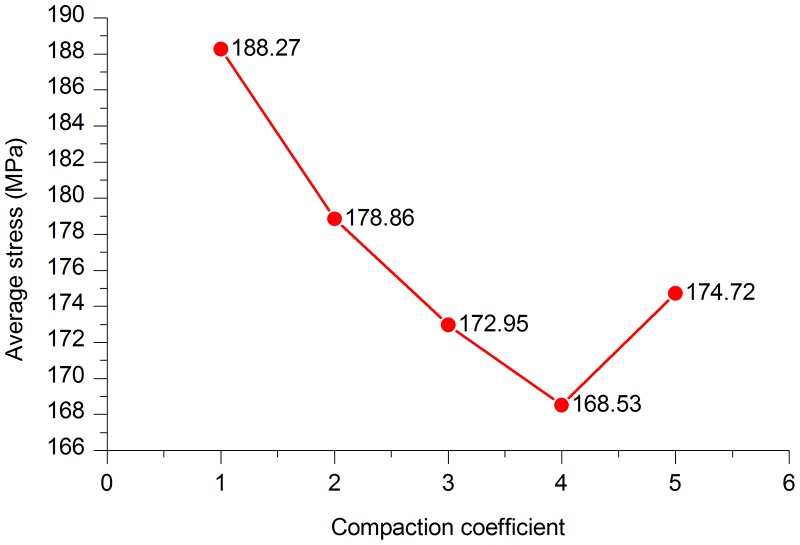
Average stress in pipe models with different Compaction coefficients.

**1. Pipe average stress**. From Fig. 16, we can conclude that the average pipe stress when compaction coefficients were 3 and 4 was lower than those when the Compaction coefficient was five, while the average pipe stresses was higher when the Compaction coefficients were 1 and 2. This means that during an earthquake, reducing soil compaction could effectively lead to the reduction of the stress of the pipeline. The reason why too small compaction caused an increase of the average stress was that the soil constraints on the pipeline cannot meet the general laying requirements.

**Fig 17:**
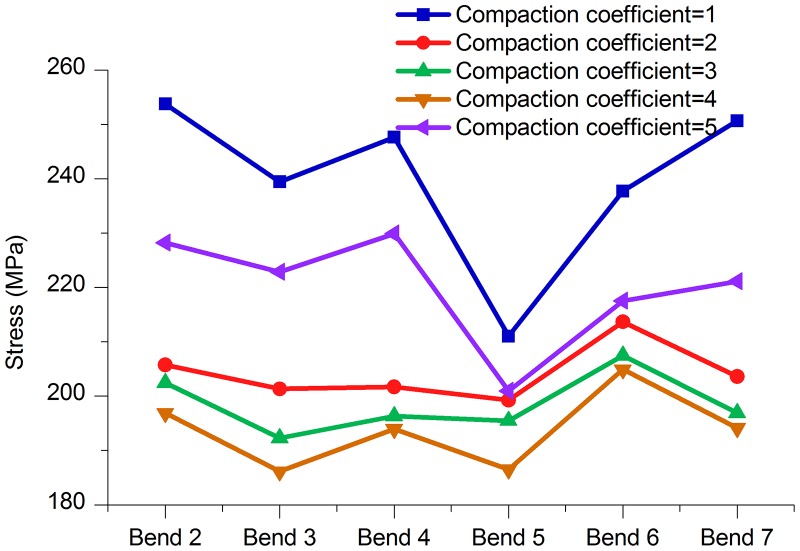
Stress of each bend with different Compaction coefficients.

**Fig 18:**
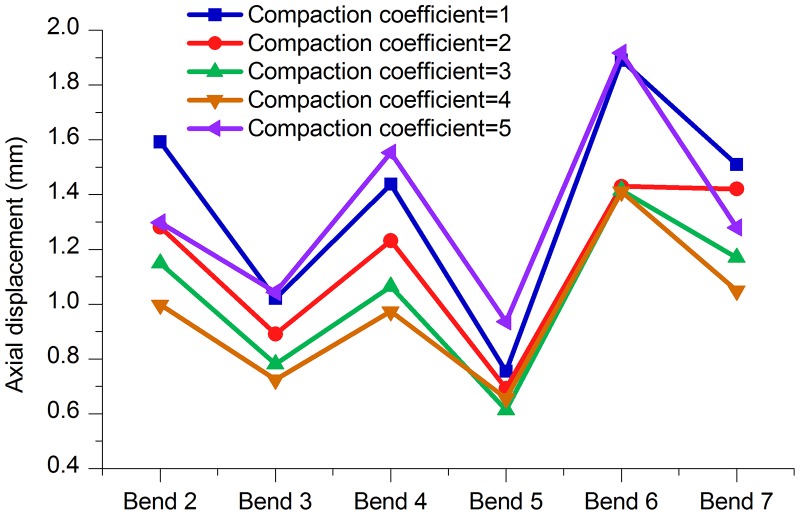
Axial displacement of bends with different Compaction coefficients.

**Fig 19:**
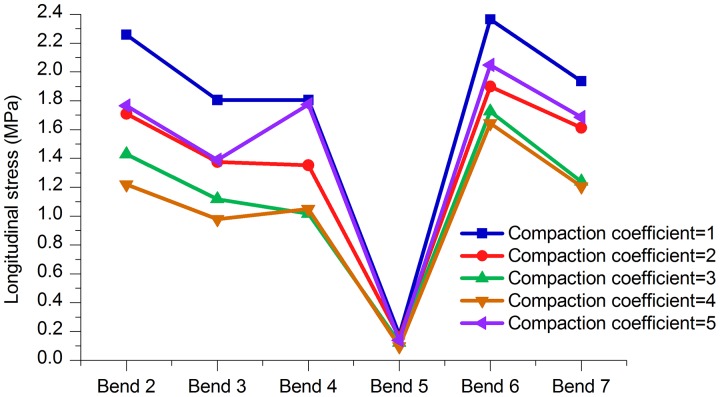
Longitudinal displacement of bends with different Compaction coefficients.

**Fig 20:**
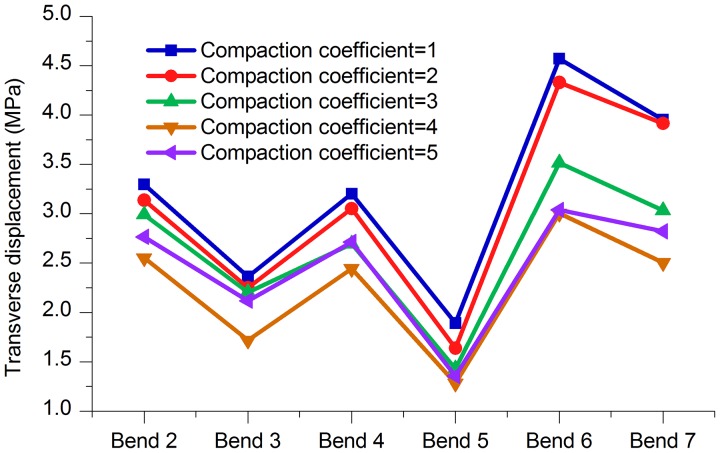
Lateral displacements of bends with different Compaction coefficients.

**2. Stress and displacement at the bends**. As shown in Fig. 17 and Table 14, the stress values of the bends in the model that was constructed when the compaction coefficients were between 2 and 4 were all smaller than when the compaction coefficient was 5. Figs. 18–20 and Tables 15–17 are axial, lateral and longitudinal displacements of bends in different pipe models built for soil models with different compaction coefficients. The comparison shows that reducing the value of Compaction coefficient, the displacement in each direction changed in a similar pattern that stresses change, i.e., all showed an increasing trend after the first decrease. In summary it can be further concluded that reducing soil compaction degree appropriately could effectively reduce stress and displacements of the pipe in various directions.

**Table 14:** Comparison of stresses at different bends (25°–45°) with different compaction coefficients.

Bend (Angle)	Compaction coefficient = 1	Compaction coefficient = 2	Compaction coefficient = 3	Compaction coefficient = 4	Compaction coefficient = 5
Bend 2 (30°)	253.80 MPa	205.71 MPa	202.41 MPa	196.87 MPa	228.22 MPa
Bend 3 (35°)	239.41 MPa	201.36 MPa	192.27 MPa	186.17 MPa	222.83 MPa
Bend 4 (35°)	247.60 MPa	201.67 MPa	196.36 MPa	194.00 MPa	229.94 MPa
Bend 5 (25°)	211.01 MPa	199.27 MPa	195.51 MPa	186.50 MPa	200.90 MPa
Bend 6 (45°)	237.73 MPa	213.67 MPa	207.50 MPa	204.90 MPa	217.56 MPa
Bend 7 (30°)	250.69 MPa	203.61 MPa	196.87 MPa	194.13 MPa	221.11 MPa

**Table 15:** Comparison of axial displacements of bends (25°–45°) with different Compaction coefficients.

Bend (Angle)	Compaction coefficient = 1	Compaction coefficient = 2	Compaction coefficient = 3	Compaction coefficient = 4	Compaction coefficient = 5
Bend 2 (30°)	1.592 mm	1.281 mm	1.150 mm	0.999 mm	1.299 mm
Bend 3 (35°)	1.020 mm	0.890 mm	0.782 mm	0.725 mm	1.044 mm
Bend 4 (35°)	1.437 mm	1.233 mm	1.064 mm	0.973 mm	1.553 mm
Bend 5 (25°)	0.755 mm	0.693 mm	0.613 mm	0.657 mm	0.936 mm
Bend 6 (45°)	1.890 mm	1.430 mm	1.416 mm	1.412 mm	1.917 mm
Bend 7 (30°)	1.510 mm	1.420 mm	1.170 mm	1.050 mm	1.280 mm

**Table 16:** Comparison of longitudinal displacements of bends (25°- 45°) with different Compaction coefficients.

Bend (Angle)	Compaction coefficient = 1	Compaction coefficient = 2	Compaction coefficient = 3	Compaction coefficient = 4	Compaction coefficient = 5
Bend 2 (30°)	2.259 mm	1.709 mm	1.430 mm	1.221 mm	1.767 mm
Bend 3 (35°)	1.805 mm	1.376 mm	1.119 mm	0.979 mm	1.391 mm
Bend 4 (35°)	1.806 mm	1.353 mm	1.017 mm	1.050 mm	1.775 mm
Bend 5 (25°)	0.176 mm	0.160 mm	0.123 mm	0.097 mm	0.138 mm
Bend 6 (45°)	2.366 mm	1.899 mm	1.725 mm	1.643 mm	2.048 mm
Bend 7 (30°)	1.935 mm	1.613 mm	1.240 mm	1.205 mm	1.686 mm

**Table 17:** Comparison of lateral displacements of bends (25°–45°) with different Compaction coefficients.

Bend (Angle)	Compaction coefficient = 1	Compaction coefficient = 2	Compaction coefficient = 3	Compaction coefficient = 4	Compaction coefficient = 5
Bend 2 (30°)	3.295 mm	3.139 mm	2.989 mm	2.554 mm	2.762 mm
Bend 3 (35°)	2.362 mm	2.250 mm	2.205 mm	1.718 mm	2.120 mm
Bend 4 (35°)	3.205 mm	3.052 mm	2.693 mm	2.443 mm	2.712 mm
Bend 5 (25°)	1.890 mm	1.635 mm	1.430 mm	1.283 mm	1.353 mm
Bend 6 (45°)	4.569 mm	4.330 mm	3.513 mm	3.006 mm	3.040 mm
Bend 7 (30°)	3.955 mm	3.913 mm	3.032 mm	2.503 mm	2.821 mm

## Conclusions

The stress analysis of an oil pipeline crossing the XX earthquake disaster area is performed. A stress analysis method and calibration standard are presented based on a frequency spectrum method. The location of the critical section and maximum stress area can be pinpointed clearly through this stress analysis. The corresponding remediation measures should be implemented based on the stress analysis.

Based on the limit-state design thinking for strength, the seismic activity with a smaller probability but severe destructive effect is loaded on the pipeline. Analysis of the stress to the XX oil transportation pipeline confirms that the bent section of the pipeline is the critical section, particularly the bended pipe at the top and bottom of the slope. The maximum axial, transverse and longitudinal displacements are generated at the bend in the pipe at the top of the slope. The earthquake activity in the axial direction poses a greater influence on the axial, longitudinal and transverse displacements. The following recommendation is presented based on the analysis: during the construction process in earthquake disaster areas, if the displacement will approach or exceed the allowable standard when the pipeline is in operation, then it is imperative to install the necessary pipe parts and shock absorbers to control the displacement of pipeline.

In addition, our discussion about the bend angle and degree of soil compaction as the dependent variables led to the conclusion that: (1) reducing the bend angle could reduce the stress and displacement of the underground pipe during an earthquake, and “Ladder” could be adopted for laying pipes; (2) appropriately reducing the degree of soil compaction could reduce the impact of the earthquake on the stress and displacement.

The proposed calibration standard, frequency analysis method and working condition loading for earthquake stress on a pipeline provides a safety reference for the design of an oil pipeline crossing an earthquake disaster area. This also fills the technology gap for the stress analysis of an oil transportation pipeline in seismic disaster locations.
